# 

**Published:** 1861-06

**Authors:** 


Published Monthly, at $3 per Annum in Advance.

### ATLANTA MEDICAL AND SURGICAL JOURNAL.

J. G. WESTMORELAND, M. D.,
PROFESSOR OF MATERIA MEDICA AND MEDICAL JURISPRUDENCE IN THE ATLANTA MEDICAL COLLEGE,
EDITOR AND PROPRIETOR.

Pax et scientia, sed veritas sine timore.

VOL. VI. JUNE, 1861. No. X.

ATLANTA, GA:

ATLANTA INTELLIGENCER PRINT.

1861.

